# Special Issue “Applications of Stable Isotope Analysis”

**DOI:** 10.3390/molecules27217293

**Published:** 2022-10-27

**Authors:** Antonio V. Herrera-Herrera, Margarita Jambrina-Enríquez

**Affiliations:** 1 Instituto Universitario de Bio-Orgánica Antonio González, Universidad de La Laguna (ULL), Avda, Astrofísico Fco, Sánchez, 2, 38206 San Cristóbal de La Laguna, Tenerife, Spain; 2 Departamento de Química, Facultad de Ciencias, Universidad de La Laguna (ULL), Avda, Astrofísico Francisco Sánchez, s/n, 38206 San Cristóbal de La Laguna, Tenerife, Spain; 3 Departamento de Biología Animal, Edafología y Geología, Facultad de Ciencias, Universidad de La Laguna (ULL), Avda, Astrofísico Francisco Sánchez, s/n, 38206 San Cristóbal de La Laguna, Tenerife, Spain

The isotopic composition of matter is controlled by different physical, chemical, and biological mechanisms. The different transformations of such matter can be examined through stable isotope analysis. Since the first stable isotope ratio measurements in 1939 by Nier and Gulbransen [1], accurate isotopic techniques have allowed the analysis of a wide variety of matrices, including environmental (e.g., sediments, soils, waters, petroleum, and meteorites), biological (e.g., bones, blood, and organic tissues), and agri-food samples (e.g., milk, oils, and wine). Thus, the application of these techniques has skyrocketed within different scientific fields such as geochemistry, archaeology, forensic science, biomedicine, environmental science, microbiology, soil science, and doping and fraud control, to name but a few. In fact, the possibilities that isotopic analysis offers are endless, and practically all disciplines of chemistry have employed these techniques.

This Special Issue aims to provide an updated view and highlight the advances in the application of stable isotope analysis (SIA) in the different fields of chemistry. It includes five research articles and two review papers dealing with the use of this technique in geochemical [2,3], archaeological [4,5,6], environmental [7], and food [4,8] studies.

The combination of SIA and stomach content analysis provides a powerful tool to assess trophic dynamics. In this regard, Chang et al. [7] used the SIA of C and N to evaluate the predatory feeding ecology of skipjack tuna in Taiwanese waters. For this purpose, the stomach contents from individuals from eastern (*n* = 347) and western (*n* = 43) Taiwan were analysed. The results of stomach contents showed that ponyfish in western Taiwan were the most important prey of tuna, while in eastern Taiwan, epipelagic squids and carangids from 2012 to 2014 and epipelagic carangids and flying fishes in 2020 were the main food items of this generalist predator. An additional Bayesian mixing model was applied, showing the importance of cephalopods and crustaceans as prey. Thanks to this study, the authors were able to conclude that skipjack tuna in western Taiwan had a slightly higher trophic position and that opportunistic predation may result in different predator–prey size dynamics between regions (stable isotope values increased with body size in eastern Taiwan). These data complemented previous research regarding other pelagic predators such as black and blue marlin, yellowfin and Pacific bluefin tuna, and sailfish in Kuroshio Current [7].

SIA in the archaeological field has evolved as a result of the close relationship of this discipline with geology and geochemistry. In this regard, Baldoni et al. [4], Skippington et al. [5] and Connolly et al. [6] applied these tools in archaeology-related studies. Baldoni et al. [4] analysed N and C isotopes to investigate the relationship between dietary habits and mortality patterns in the Roman Imperial and Medieval population of 18 archaeological sites from Italy. Their study suggested variations in survival between sexes in the Roman times (with women of childbirth age experiencing a higher mortality risk). Furthermore, N isotopic signatures seem to be related to mortality in both periods, which was shown to have a quadratic effect in the Roman Imperial period and was shown to be linear in the Middle Ages. As for C, no influence was detected in the Roman population, whereas a higher mortality risk was observed in the medieval population for increasing C isotopic values. These results were consistent with the fact that diet strongly influences populations.

The research developed by Skippington et al. [5] demonstrates that the SIA of tooth enamel from macropods (spectacled hare wallaby and hill kangaroo) can be used with confidence to trace paleoenvironmental changes in Australian archaeological contexts at inter-strata resolution. To this end, stable carbon (δ^13^C) and oxygen (δ^18^O) isotope ratios of dental carbonates from both archaeological and modern samples from Boodie Cave (Barrow Island) were analysed. The δ^18^O values followed the dynamic palaeoecological history (climatic changes) of the cave (with a shift towards increased aridity preceding the onset of the Last Glacial Maximum and a period of augmented wetness in the early-to-mid-Holocene). These corresponded with available continental and regional models. Meanwhile, enamel δ^13^C values may imply a long-term shift towards greater variety in vegetation.

Connolly et al. [6] developed experimental research to study the influence of combustion on δ^2^H_wax_ values in order to test the potential of thermally altered archaeological combustion structures as climatic tools and to link climatic and archaeological datasets at a high spatial and temporal resolution. For this purpose, a series of experiments was carried out to heat leaf, bark, and xylem tissues of the *Celtis australis* plant under oxygen-limited conditions at 150, 250, 350, and 450 °C for limited times (1 h). Compound-specific isotopic analysis (CSIA) which focused on *n-*alkanes showed that while oscillations in the δ^2^H_wax_ of leaves were minimal up to 350 °C, significant variation was observed at temperatures >450 °C and in the xylem and bark samples. Thus, in the absence of a detailed characterisation of the depositional context, the application of δ^2^H_wax_ as a palaeoclimate proxy should be cautiously interpreted when applied to anthropogenic environments.

The article by Bianchini et al. [8] deepens our understanding one of the typical applications of SIA in food sciences: authentication. Considering that the isotopic composition of a particular food, despite metabolic fractionation, is related to the ecosystem, trophic level and food chain, the authors used elemental analysis coupled to isotope ratio mass spectrometry to identify the isotopic fingerprints of clams from Adriatic lagoons. The use of C/N, δ^13^C, and δ^15^N in tissues; δ^13^C in shells; and Δ^13^C (calculated as δ^13^C_shell_ − δ^13^C_tissues_) allowed local clams to be distinguished from products from other parts of the world (δ^13^C and δ^15^N in tissues) and the point of origin of the local ones (the isotopic fingerprints of shells, C/N, and the isotopic compositions of tissues). The authors also indicated that further studies based on the temporally simultaneous collection of samples and the incorporation of other isotopic parameters are needed to obtain a better tool for the authentication of origin and provenance.

Finally, the works by Ogbesejana et al. [2] and Nyamgerel et al. [3] reviewed different aspects related to the applications of geochemistry, due to the recurrent use of SIA in this discipline. Ogbesejana et al. [2] reviewed the SIA of organic elements within shales and crude oils. Due to the usefulness of this kind of analysis in petroleum geochemistry, the bulk and CSIA of S, C, and H as well as their relationships with the source, depositional environments, thermal maturity, geological age, oil–oil and oil–source rock correlation, and gas exploration were the subject of the study. In addition, instrumental considerations of the different approaches were also discussed. The authors were able to conclude that the number of studies addressing the use of sulphur isotopic compositions of biomarkers in the crude oil and CSIA of shale are very limited due to the relatively low concentrations of these compounds (and the subsequent absence in regular gas chromatograms). For this reason, more routine molecular sieving methods are needed to preconcentrate these compounds, and the use of modern isotopic techniques (i.e., laser microprobe, ion microprobe, and NanoSIMS) is recommended for studies regarding shales and crude oils.

The article by Nyamgerel et al. [3] reviews the use of ^17^O as a new tracer within the triple oxygen isotope system (^16^O, ^17^O, and ^18^O) due to its usefulness in hydrological and climatological investigations (related to its sensitivity to environmental conditions). Recent analytical developments to measure small variations in ^17^O (primarily dual-inlet isotope ratio mass spectrometry and laser absorption spectroscopy) have facilitated such studies, providing information regarding atmospheric conditions at the moisture source and isotopic fractionations during transport and deposition processes. Variations in δ^17^O with respect to the developed global meteoric water line have demonstrated the importance of regional or local effects on ^17^O distribution. A wide variety of publications have used these isotopes for diverse purposes, including the detection of changes in moisture origin, the mixing of different water vapours, evaporative loss in dry regions, the re-evaporation of raindrops during warm precipitation, and convective storms in low- and mid-latitude waters. However, due to the large variation in the spatial scale of hydrological processes with extent, additional studies and in situ measurements are needed to facilitate accurate and robust simulations.

Despite the fact that the quality of isotopic measurements is highly dependent on different critical factors (thus requiring specialised instrumentation and staff), we truly believe that this collection is highly attractive to the research community due to the increasing interest in isotopic analysis.
